# Mechanistic Study of Manganese-Substituted Glycerol Dehydrogenase Using a Kinetic and Thermodynamic Analysis

**DOI:** 10.1371/journal.pone.0099162

**Published:** 2014-06-04

**Authors:** Baishan Fang, Jin Niu, Hong Ren, Yingxia Guo, Shizhen Wang

**Affiliations:** 1 Department of Chemical and Biochemical Engineering, College of Chemistry and Chemical Engineering, Xiamen University, Xiamen, China; 2 The Key Lab for Synthetic Biotechnology of Xiamen City, Xiamen University, Xiamen, China; University of Alberta, Canada

## Abstract

Mechanistic insights regarding the activity enhancement of dehydrogenase by metal ion substitution were investigated by a simple method using a kinetic and thermodynamic analysis. By profiling the binding energy of both the substrate and product, the metal ion's role in catalysis enhancement was revealed. Glycerol dehydrogenase (GDH) from *Klebsiella pneumoniae* sp., which demonstrated an improvement in activity by the substitution of a zinc ion with a manganese ion, was used as a model for the mechanistic study of metal ion substitution. A kinetic model based on an ordered Bi-Bi mechanism was proposed considering the noncompetitive product inhibition of dihydroxyacetone (DHA) and the competitive product inhibition of NADH. By obtaining preliminary kinetic parameters of substrate and product inhibition, the number of estimated parameters was reduced from 10 to 4 for a nonlinear regression-based kinetic parameter estimation. The simulated values of time-concentration curves fit the experimental values well, with an average relative error of 11.5% and 12.7% for Mn-GDH and GDH, respectively. A comparison of the binding energy of enzyme ternary complex for Mn-GDH and GDH derived from kinetic parameters indicated that metal ion substitution accelerated the release of dioxyacetone. The metal ion's role in catalysis enhancement was explicated.

## Introduction

Various metalloenzymes act in fundamental biological processes found in nature. The metal ion of most metalloenzymes participates in the catalytic process involved in the function of the polarization of chemical bonds, nucleophile activation, and substrate or product coordination [1]. Substitution of metal ions in metalloenzymes is a mild and effective modification that is used in structure-function relationship studies [2]. Metal ion substitutions have been reported to change the catalytic activity [3], substrate specificity [4], and stability [5], [6] of metalloenzymes and have stimulated much research interest. The mechanism of metal ion substitution has been mainly studied by molecular simulation and the analysis of substituted enzyme crystals. Sparta [7] studied the metal ion substitution effects of catechol-O-Methyltransferase activity by a quantum mechanical/molecular mechanical dynamics method. Metal ion substitution affected the rate-limiting step, which was explicated as the methyl transfer that occurred with a significant increase in the activation barrier. D'Antonio investigated the structure of cobalt-reconstituted human arginase I, revealing the change of the catalytic mechanism upon metal ion substitution [8]. However, the molecular simulation of the enzyme structure and the generation of metal ion substituted enzyme crystals are lengthy and expensive processes.

Compared with dynamic simulation and structure analysis, thermodynamic and kinetic analysis is a simple, quick and valid strategy aimed at explaining the activity and functional properties of biocatalysis systems [9]. The microenvironment of the active site of the metalloenzyme involved in the reaction mechanism undergoes dynamic and structural changes upon metal ion substitution [10]. Free energy binding profiles of substrates and products with modified enzymes and enzyme-substrate complexes, which demonstrate the catalytic requirement for transition state stabilization and ground state stabilization, offer an alternative method for studying the influence of metal ion substitution on catalytic reactions [11]. A kinetic and thermodynamic analysis of each binding step of the substrate and product can be used for profiling the significant changes caused by metal ion substitution and can provide valuable information for further study.

Glycerol dehydrogenases play crucial roles enzymes in the pathway of glycerol metabolism, industrial applications and even pathogenicity [12], [13]. The NAD^+^-linked GDHs are members of the medium-chain alcohol dehydrogenase family, most of which are metalloenzymes [14]. They dehydrogenate glycerol to dihydroxyacetone and lead to the production of value-added products, namely, DHA, butanol, succinic acid and citric acid [15], [16]. GDHs are also widely used for the enzymatic determination of glycerol for medical diagnoses and fermentation process analyses [17], [18]. Coupling with other oxidoreductases, GDH is part of a multi-enzyme system for the biosynthesis of chiral intermediates with cofactor regeneration [19]. Therefore, glycerol dehydrogenase was selected as a model enzyme for this metal ion substitution study.

Glycerol dehydrogenase from *Klebsiella pneumoniae* sp. is a zinc-dependent metalloenzyme [20]. It has been previously shown that Mn^2+^ substituted GDH exhibits improved activity and thermostability [21]. In this paper, mechanistic insights of activity improvement are studied based on kinetic and thermodynamic analysis. A kinetic model based on an ordered Bi-Bi mechanism with substrate and product inhibition is proposed. The equilibrium constants for each ligand-binding are calculated by using the forward and reverse rate constants. By profiling the binding rate and energy for substrate and product with enzyme, the rate accelerating step is determined. The metal ion's role in catalysis enhancement is investigated.

## Results and Discussion

### The influence of substrate concentration

**Figure 1:**
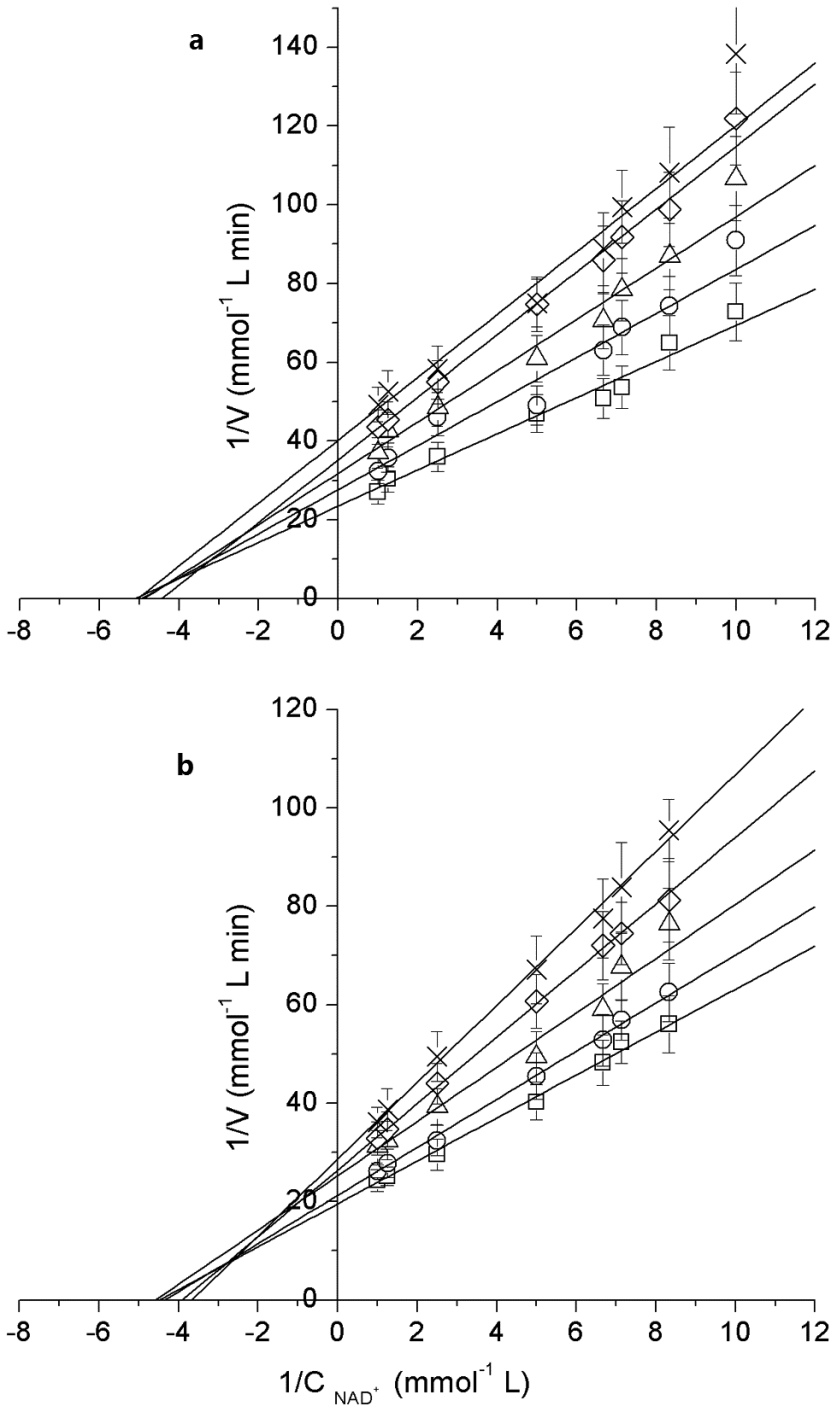
The influence of glycerol concentration. a. The influence of glycerol concentration for GDH; b. The influence of glycerol concentration for Mn-GDH. Reaction conditions: Glycerol concentration (×: 0.010 mol/L, 0.015 mol/L, Δ: 0.025 mol/L, O: 0.100 mol/L, □: 0.200 mol/L); enzyme, 1 mg/L; pH 12.0; temperature, 45°C.

The influence of substrate concentration of both substrates, NAD^+^ and glycerol, on GDH (Fig. 1a) and Mn-GDH (Fig. 1b) were studied. Double reciprocal plots of six NAD^+^ concentrations versus reaction rates at six fixed glycerol concentrations were drawn [22]. Earlier studies that were conducted on glycerol dehydrogenase from *Klebsiella pneumonia* [23] and from other microorganisms [24], [25] reported the GDHs follow an ordered Bi-Bi sequential mechanism. Therefore, it was reasonable to assume that GDH from *Klebsiella pneumonia* and Mn-GDH obey the ordered Bi-Bi sequential mechanism. Kinetic parameters were determined from Lineweaver-Burke plots. The kinetic parameters for GDH and Mn-GDH are listed in Table 1.

**Table 1:** Kinetic parameters of Mn-GDH and GDH.

Kinetic parameters	Mn-GDH	GDH
*K_mA_* * (mmol/L)	0.008±0.001	0.005±0.001
*K_mB_* *(mmol/L)	0.226±0.013	0.262±0.014
*K_iA_* (mmol/L)	0. 012±0.002	0.008±0.001

*K_mA_, the Michaelis-Menten constant of NAD^+^; *K_mB_*, the Michaelis-Menten constant of glycerol.

Reaction conditions: Glycerol concentration (0.010–0.400 mmol/L); enzyme, 1 mg/L; pH 12.0; temperature, 45°C.

### The influence of product concentration

**Figure 2:**
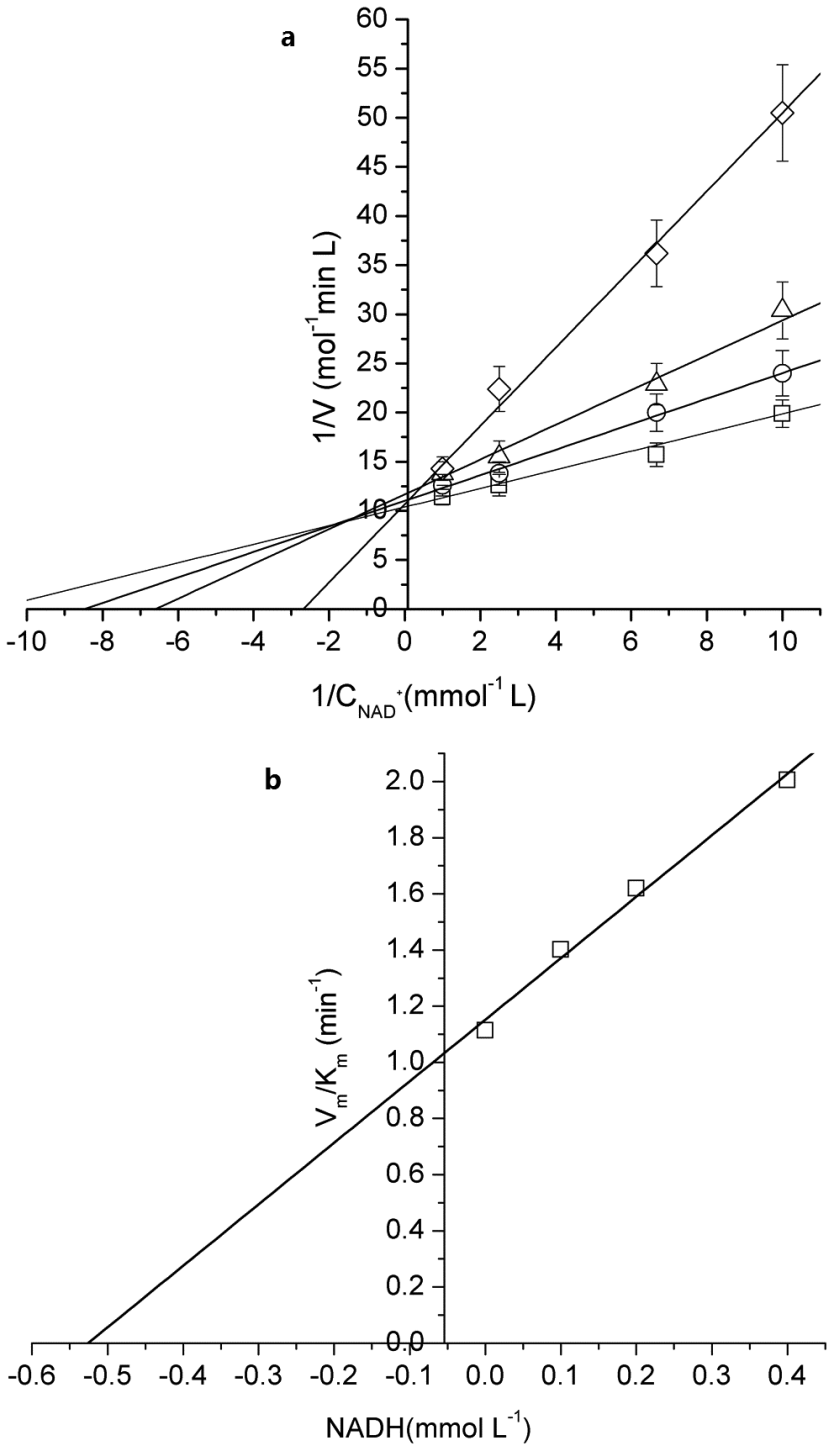
Product inhibition of NADH for Mn-GDH. a. Double reciprocal plots of NADH concentrations versus reaction rates b. The secondary plots of the slopes for product inhibition of NADH for Mn-GDH. Reaction conditions: NADH concentration (□: 0 mmol/L, Ο: 0.05 mmol/L, Δ: 0.10 mmol/L, ◊: 0.20 mmol/L); glycerol 0.40 mol/L; Mn-GDH, 1 mg/L; pH 12.0; temperature, 45°C.

The product inhibition of Mn-GDH was studied by varying the concentration of each product, DHA (0–0.40 mmol/L) and NADH (0–0.20 mmol/L). NADH competitively inhibited the enzyme at a constant concentration of glycerol (Fig. 2a), which was consistent with a compulsory ordered Bi-Bi reaction mechanism. The inhibition constant of NADH, *K*_iQ_, which was derived by secondary plots (Fig.2b) of the slopes determined from the primary double-reciprocal plots [22] against each fixed NADH concentration, was calculated as 0.02 mmol/L.

**Figure 3:**
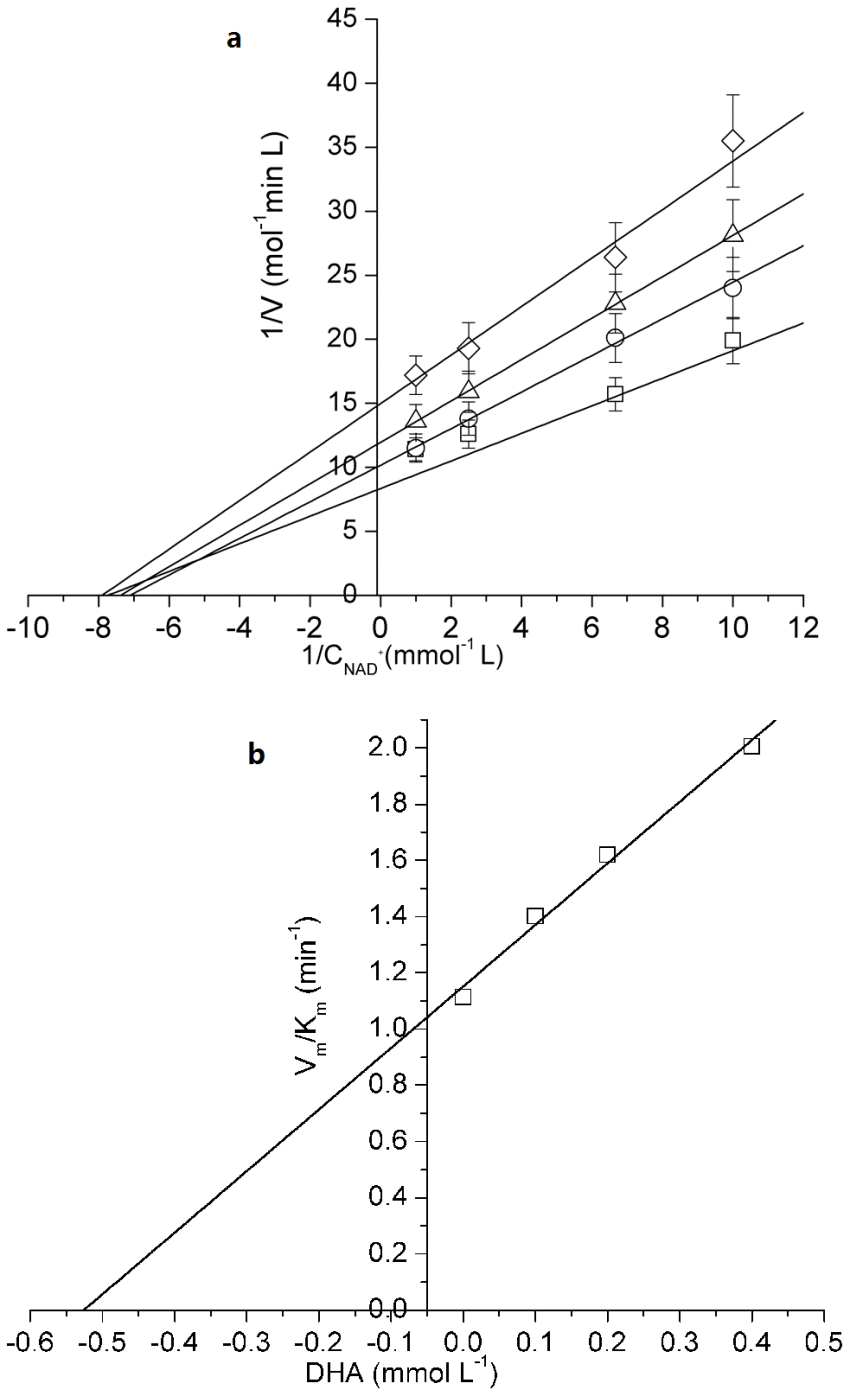
Product inhibition of DHA for Mn-GDH. a. Double reciprocal plots of NADH concentrations versus reaction rates. b. The secondary plots of the slopes for product inhibition of DHA for Mn-GDH. Reaction conditions: DHA (□: 0 mmol/L, Ο: 0.10 mmol/L, Δ: 0.20 mmol/L, ◊: 0.40 mmol/L); glycerol 0.40 mol/L; Mn-GDH, 1 mg/L; pH 12.0; temperature, 45°C.

DHA inhibited the enzyme noncompetitively with respect to NAD^+^ at a constant concentration of glycerol (Fig. 3a). The inhibition constant of DHA, *K_iP_*, derived by secondary plots of the slopes determined from the primary double-reciprocal plots (Fig.3b), was calculated as 0.52 mmol/L. Product inhibition indicated that Mn-GDH obeyed a compulsory ordered-Bi-Bi mechanism. The inhibition constant of DHA and NADH for GDH were calculated. *K_iP_*, and *K_iQ_* were 0.45 mmoL/L and 0.015 mmoL/L, respectively.

### Kinetic model development

**Figure 4:**
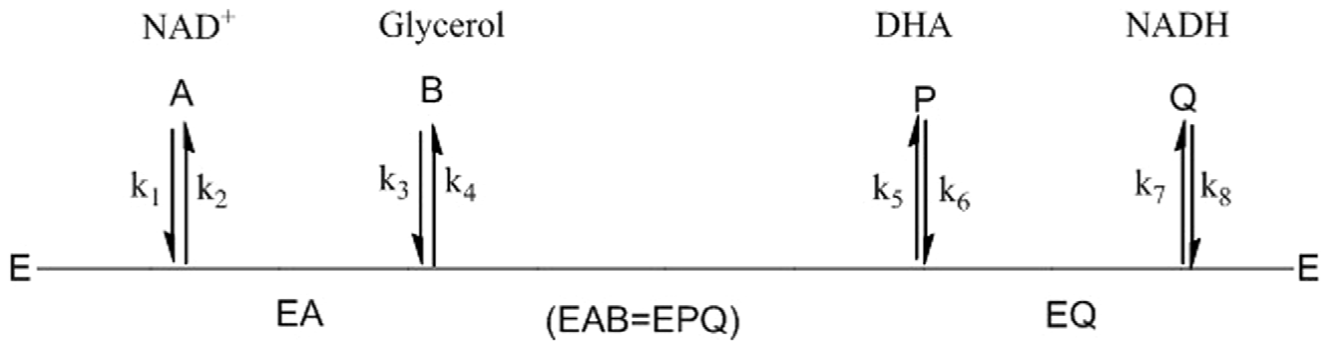
Compulsory ordered Bi-Bi reaction mechanism.

An ordered Bi-Bi mechanism kinetic model was proposed. The King-Altman plot of this model is shown in Fig. 4. In this model, NAD^+^ (A) binds first to the free enzyme (E). The second substrate, glycerol, binds subsequently, forming the ternary complex (EAB). Upon isomerization, the product-bound complex (EPQ) is formed. After the release of the first product, DHA (P), the second product, NADH (Q), dissociates, leaving the free enzyme.

The corresponding volumetric rate of NADH, derived from King-Altman method and transformed using Cleland's coefficient form, is given by Eq.1.
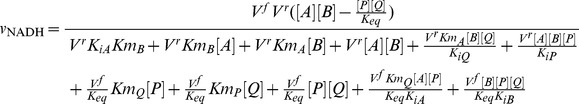
(1)


Eq.2 and Eq.3, which describe the relevance between kinetic constants, were obtained by applying Haldane equations. *K_eq_* and *K_iB_* were replaced with Eq.2, Eq.3, respectively. The value of *K_mA_*, *K_mB_*, *K_iA_*, *K_iP_* and *K_iQ_* were derived from the above experiments. 
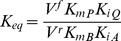
(2)

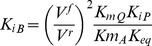
(3)


### Parameter estimation

**Figure 5:**
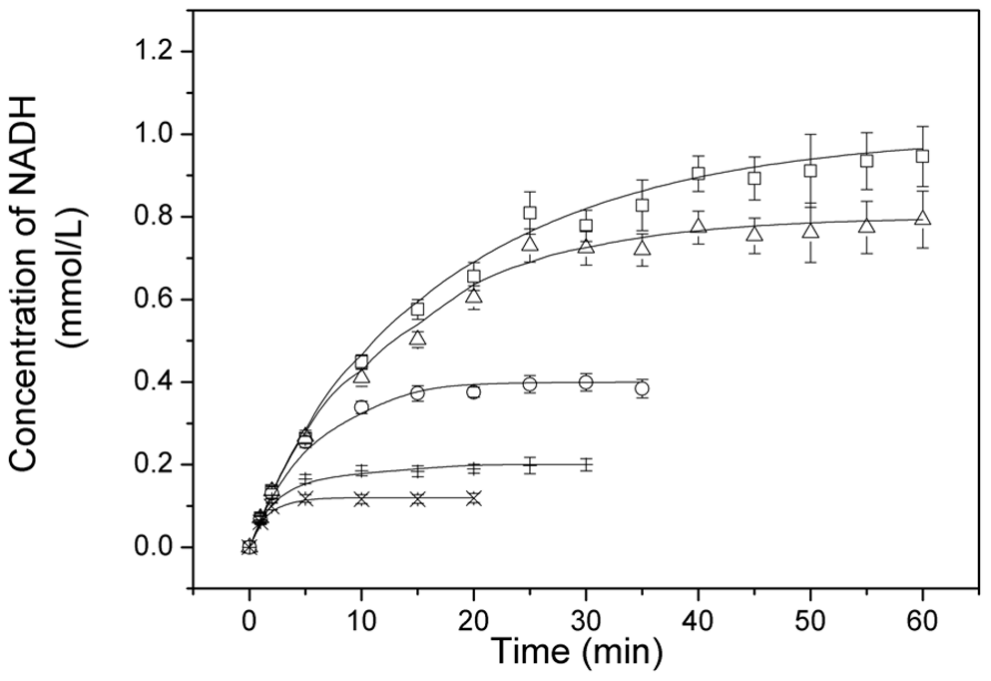
Comparison of the simulated values with the experimental data for Mn-GDH reaction conditions: NAD^+^ (□: 1.0 mmol/L; Δ: 0.8 mmol/L, Ο: 0.4 mmol/L, +: 0.2 mmol/L, ×: 0.12 mmol/L), simulated (lines, —); glycerol, 0.1 mol/L; Mn-GDH, 1 mg/L; pH, 12.0; temperature, 45°C.

**Figure 6:**
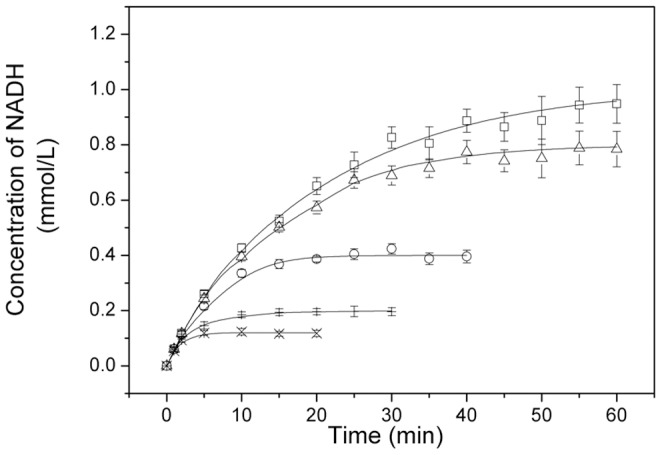
Comparison of the simulated values with the experimental data for GDH. reaction conditions: NAD^+^ (□: 1.0 mmol/L; Δ: 0.8 mmol/L, Ο: 0.4 mmol/L, +: 0.2 mmol/L, ×: 0.12 mmol/L), simulated (lines, —); glycerol, 0.1 mol/L; GDH, 1 mg/L; pH, 12.0; temperature, 45°C.

The values of the kinetic parameters, namely *K_mA_*, *K_mB_*, *K_iA_*, *K_iB_*, *K_iP_*, *K_iQ_*, was obtained previously, which were used for the final parameter estimation via nonlinear regression [26]. *K_eq_* is a dependent parameter. Therefore, the number of estimated parameters was reduced from 10 to 4. Kinetic parameters for estimation can be remarkably decreased. The concentration-time curve data with various substrate concentrations were used for the simulation of the remaining four parameters, namely, *V^f^*, *V^r^*, *K_mP_*, and *K_mQ_*, by the MATLAB program. Parameter estimation was carried out by a combination of fourth- and fifth-order Runge kutta method using ode45 module in MATLAB software. All datasets were fitted at once. These parameters are listed in Table 2. The comparisons of simulated values with the experimental data for Mn-GDH and GDH are shown in Fig. 5 and Fig. 6. There was a good agreement between the experimental and simulated values, with 11.5% and 12.7% average relative error for Mn-GDH and GDH, respectively.

**Table 2:** Estimated values of kinetic parameters.

Kinetic parameters	Mn-GDH	GDH
*V^f^* (mmol/L/min)	0.080	0.068
*V^r^* (mmol/L/min)	0.0104	0.0074
*K_mA_* *(mmol/L)	0.008	0.262
*K_mB_* * (mmol/L)	0.005	0.226
*K_iA_* * (mmol/L)	0.012	0.008
*K_iB_* ** (mmol/L)	0.301	0.66
*K_mP_* (mmol/L)	0.451	0.28
*K_mQ_* (mmol/L)	0.002	0.0016
*K_iP_* * (mmol/L)	0.52	0.45
*K_iQ_* * (mmol/L)	0.02	0.015
*K_eq_* **	25.6	18.4

*: the fixed parameters.

**: the dependent parameters.

### Thermodynamic study

The thermodynamic parameters, which are highly complex and interdependent, can be derived from kinetic parameters. Most ligand-binding reactions are studied by calculating the values of the binding equilibrium constant and binding energy of each step [11]. Parameters for each step provide further insights into the binding process, which reveals the effect of metal ion substitution.

The rate constants for each binding step (*k_1_-k_8_*), which are shown in Table 3, were calculated according to the definition of rate constants in terms of kinetic constants [22]. *K_EA_*, *K_EAB_*, *K_EPQ_* and *K_EQ_* were assigned as the equilibrium constants for each ligand-binding event [11]. The binding equilibrium constants are listed in Table 4. The Mn-GDH and GDH have nearly the same *K_EAB_* value, while a higher *K_EPQ_* value for Mn-GDH.

**Table 3:** The rate constants.

Rate constants	Mn-GDH	GDH
*k_1_* (L/min/mmol)	10000	13600
*k_2_* (/min)	120	109
*k_3_* (L/min/mmol)	366	260
*k_4_* (/min)	11	8
*k_5_* (/min)	347	3431
*k_6_* (L/min/mmol)	725	11446
*k_7_* (/min)	104	69
*k_8_* (L/min/mmol)	5200	4625

**Table 4:** The binding equilibrium constants of enzyme-substrate complex.

Binding equilibrium constants	Mn-GDH	GDH
*K_EA_*	0.012	0.008
*K_EAB_*	0.030	0.030
*K_EPQ_*	0.48	0.30
*K_EQ_*	0.02	0.015

Binding energy of the enzyme ternary complex EPQ (enzyme-DHA-NADH complex), which were defined as -RTln*K_EPQ_*, for Mn-GDH and GDH were 1.95 and 3.18 KJ/mol, respectively. This revealed that the substitution of manganese mainly depended on accelerating the release of dioxyacetone [27], [28].

Manganese has the similarity with the other divalent cations, which are Lewis acids and electrostatic stabilizers. Manganese can be replaceable with other metals, namely, magnesium and zinc. This depends on the intermediate properties of Mn^2+^ relative to these other ions, including its radius length and borderline hard–soft character [29]. Metal ions with important biological function can be classified as hard and soft, while hard acids and bases are weak polar, have small ionic radius and high oxidation state. The soft species are on the contrary [30]. Replacing the catalytic zinc with manganese revealed electronic requirements for the specific geometry of the catalytic site of GDH.

## Conclusion

A simple, quick and valid strategy based on kinetic and thermodynamic analysis for understanding the significant mechanistic changes induced by metal ion substitution was proposed. By profiling the binding rate and energy for the substrate and product of the enzyme, the rate accelerating step was determined. The mechanism of increasing the catalytic activity of glycerol dehydrogenase by manganese substitution was studied. A kinetic model based on the ordered Bi-Bi mechanism with both product and substrate inhibition was proposed. Further mechanistic insights regarding the role of the metal ion in catalysis enhancement were investigated by characterizing the binding kinetics and thermodynamics of each step. The kinetic parameters were simulated by the MATLAB program. The thermodynamic parameters were derived from the kinetic parameters, which indicated that the metal ion substitution facilitated the release of dioxyacetone.

The kinetic and thermodynamic analysis contributed further insights regarding the prediction and optimization of polyol dehydrogenases that are widely used for chiral alcohol production. This study also provided valuable information for further investigation by molecular simulation. Metal coordination is a key structural and functional component of enzyme. Given this dual role, metal coordination plays a template role in folded and functional protein domains and complexes. The study of metal ions coordination effect based on kinetic and thermodynamic study will provided a promising method metal induced multi-enzyme assembly study [31].

## Materials and Methods

### Materials

Glycerol, NAD^+^, NADH, and ethylenediaminetetraacetic acid (EDTA) were purchased from Sigma Chem. Co. (Beijing, China). The media (Tryptone, yeast extract, nutrient broth) were purchased from Sangon Biotech Co. Ltd. (Shanghai, China). All other chemicals used were analytical grade and were purchased from either Sigma or Merck (Beijing, China).

### Preparation of the apoenzyme and metal substitution

The preparation of purified GDH followed a previously published method [19]. Recombinant GDH was expressed by transforming plasmids containing the *gld*A gene (GenBank: AKAM01000021.1), which codes for glycerol dehydrogenase, into *E. coli* BL21 (DE3). Metal substitution was accomplished using purified GDH using the following steps: chelation of GDH's catalytic zinc ion using EDTA (1.0 mmol/L) for 4 h at 28°C and removal of EDTA-Zn^2+^ from the GDH solution by dialysis in binding buffer (pH 7.4, changed every 8 h) at 4°C for 53 h. The manganese ion was introduced by the co-incubation of the GDH apoenzyme with MnCl_2_ (10.0 mmol/L) for 1 h at 37°C. A reference experiment was performed by directly adding Mn^2+^ (10 mmol/L) to native GDH to investigate the activation effects of bivalent ions. The results indicated that the addition of Mn^2+^ to the reaction solution decreased the activity of GDH. The activity of the apoenzyme after EDTA treatment was nearly undetectable, which confirmed the activity enhancement by metal ion substitution [21].

### Enzyme assay and kinetic study

The activity of glycerol dehydrogenase was measured by following the increase of NADH concentration using a Spectra Max M5 Microplate Reader (California, United States). Measurements were taken at 340 nm, and a molar extinction coefficient of 6.22/mM/cm was used. One unit of GDH activity was defined as the amount of enzyme necessary to oxidize 1 µmol of NADH per minute under the following conditions (45°C, 0.1 mol/L potassium carbonate buffer, pH 12.0). The assay mixture contained 0.4 mol/L glycerol, 0.1–1.0 mmol/L NAD^+^, and 0.1 mol/L potassium carbonate buffer (pH 12.0). The volume of the reaction mixture was 200 µL in all cases. Reactions were started by the addition of the enzyme solution. Enzyme activities were determined in triplicate. Three blank controls were used as a reaction mixture with the apoenzyme, without the enzyme, and without NAD^+^. For the product inhibition study, the reactions were carried out with different DHA or NADH concentrations.
